# Vision of the first EPMA center for predictive, preventive and personalized medicine in Europe

**DOI:** 10.1186/1878-5085-5-S1-A153

**Published:** 2014-02-11

**Authors:** Marko Kapalla, Juraj Kubáň, Vincenzo Costigliola, Olga Golubnitschaja

**Affiliations:** 1 Institute of Medical Chemistry, Biochemistry and Clinical Biochemistry, Faculty of Medicine, Sasinkova 2, Comenius University, 811 08 Bratislava, Slovakia; 2 Negentropic Systems, Murgasova 12, 03401 Ruzomberok, Slovakia; 3 European Association for Predictive, Preventive and Personalised Medicine (EPMA), Avenue des Volontaire 19, 1160 Brussels, Belgium; 4 Department of Radiology, Rheinische Friedrich-Wilhelms-University of Bonn, Germany

## Introduction

Paradoxically, despite scientific and technological progress over the last decades, health of an individual person is under perpetual attack from all types of external and internal factors. On one hand, life expectancy is increasing, on the other hand the quality of life at higher age is rather questionable even in developed countries and as a result people spend many years of their lives by visiting their physicians and hospitals instead of enjoying life with their families and friends. On global scale, systems of healthcare spend enormous amount of money (7-12 % of GDP, approx. 7 trillion USD was spend worldwide on healthcare in 2011) [1] to cover treatment of mostly preventable diseases instead of changing philosophy from collapsing and unbalanced **disease-oriented healthcare**, which we refer to as “diseasecare”, to the **health-oriented healthcare** as an economically sustainable, logical, and progressive alternative and inevitable balance.

## The vision of PPPM Center

**Figure 1:**
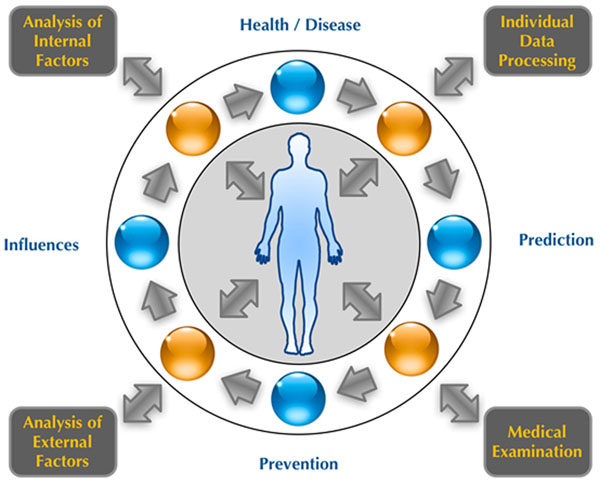
Block scheme of the synergies of technologies engaged in the envisioned PPPM center.

Predictive, preventive and personalized medicine (PPPM) represents twilight of the new era in medicine of the 21^st^ century [2] and leads us to the vision of building a novel type of a complex healthcare facility consisting of several entangled units such as medical unit, clinical laboratories, imaging techniques, bioinformatics and specific computer models [3,4], healthy nutrition, analytical laboratories, and other health-related synergy units (Fig. 1) with integrated components that are essential for the evidence-based early prediction of the health problems, preventive action and personalized attitude in apparently healthy individuals who care about their own health in order to avoid the state of ever being called “a patient”. PPPM Center, visioned as an ecosystem utilizing renewable resources, will offer an excellent opportunity for an interdisciplinary cooperation and health-related frontier science research and its real-time application in healthcare. The center will support innovation, open mind, invention, expertise, international cooperation, ethics and **networking with established facilities sharing the same values**. Supported by the **new EU legislation**, it will be aimed at promoting PPPM and all related technologies and philosophies, such as integrative medicine, technological medicine, complementary and alternative medicine, laboratory medicine, knowledge mining, healthy life style, health education, to name but a few that EPMA finds essential for healthcare of the 21^st^ century.

The international network of PPPM centers will create a backbone of the health-oriented healthcare and would represent a parallel to the hospitals, clinics and other disease-oriented facilities which current healthcare is based on. While serving for the benefit of the individual clients, the center will, undoubtedly, bring benefits also for the whole society. From economics point of view, success of such a pioneering center paves the way for the reliable investment in future healthcare and **may trigger other related investments and spin-off projects which may stimulate economic growth and help to overcome the current crisis.**

## Shared values and recommendations

**Figure 2:**
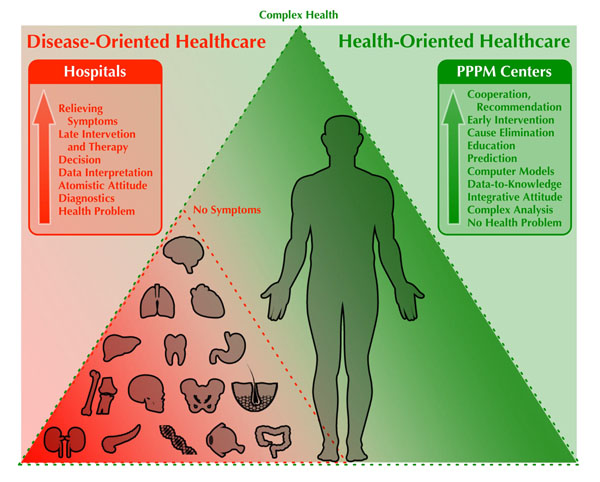
Visualization of the philosophy and the role of PPPM centers in health-oriented healthcare. Both systems should cooperate and point towards complex health of each individual person.

Current disease-oriented healthcare is gradually and unequivocally lacking the very essence of what the healthcare and medicine should be here for – helping people stay in good physical, mental and emotional health throughout the lifetime. We believe that the quality of life at all levels can be substantially increased through the realization of the vision above for it relates not only to technology and healthcare services but it touches the very basic question of the lifetime philosophy as well as the “spiritual health” which is largely being destroyed by current western “civilization” and its attitude towards health of every individual person. The basic role of PPPM centers and the philosophy in health-oriented healthcare is outlined in (Fig. 2).

With respect to the outlined vision EPMA experts recommend the following:

1. new EU legislation recognizing PPPM as an essential component of the health-oriented healthcare which cooperates with disease-oriented healthcare in order to help any and every individual person to stay healthy or gain health as soon as possible

2. creating network of specialized PPPM centers under EPMA umbrella

3. financial support for specialized PPPM healthcare centers and their networking

4. supporting health-related education in all EU countries

5. supporting investors willing to participate in creating new healthcare
